# Supplementary material for: The causal relationship between the human gut microbiota and pyogenic arthritis: a Mendelian randomization study
Source: Front Cell Infect Microbiol. 2024 Nov 26;14:1452480. doi: 10.3389/fcimb.2024.1452480 (PMC11629706; doi:10.3389/fcimb.2024.1452480)
Supplement: Supplementary file 1 [file DataSheet1.zip › mendelian test/ebi-a-GCST90027749.csv_leave_one_out.pdf]

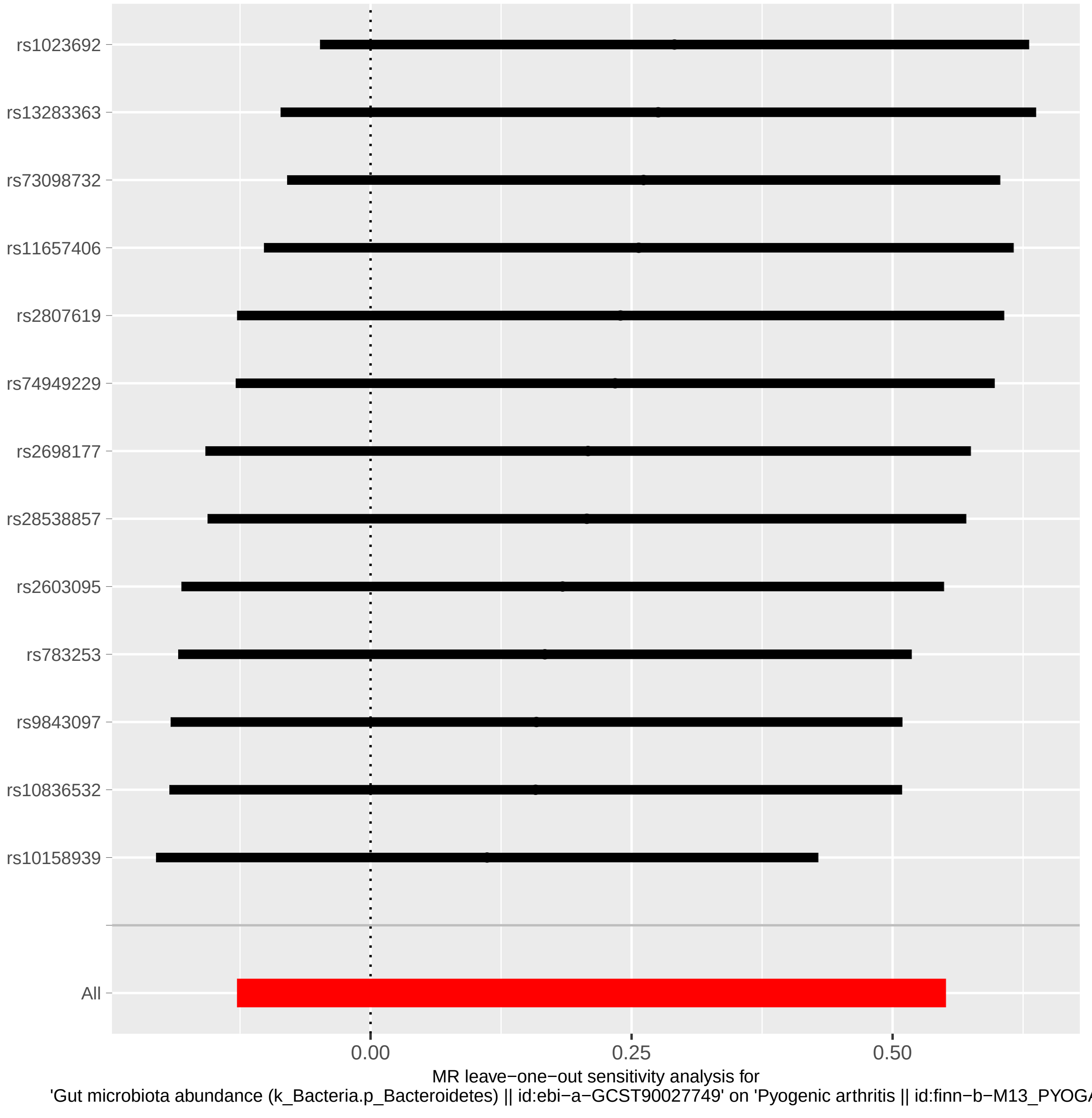
rs1023692
rs13283363
rs73098732
rs11657406
rs2807619
rs74949229
rs2698177
rs28538857
rs2603095
rs783253
rs9843097
rs10836532
rs10158939
All
0.00
0.25
0.50
MR leave−one−out sensitivity analysis for
'Gut microbiota abundance (k_Bacteria.p_Bacteroidetes) || id:ebi−a−GCST90027749' on 'Pyogenic arthritis || id:finn−b−M13_PYOG
